# Lysine-specific histone demethylase 1B (LSD2/KDM1B) represses p53 expression to promote proliferation and inhibit apoptosis in colorectal cancer through LSD2-mediated H3K4me2 demethylation

**DOI:** 10.18632/aging.103558

**Published:** 2020-07-29

**Authors:** Shaoxin Cai, Jinsi Wang, Wei Zeng, Xuefei Cheng, Lihang Liu, Weihua Li

**Affiliations:** 1 Shengli Clinical Medical College of Fujian Medical University, Fuzhou 350001, China; 2 Department of Surgical Oncology, Fujian Provincial Hospital, Fuzhou 350001, China

**Keywords:** colorectal cancer, proliferation, apoptosis, LSD2, p53

## Abstract

Epigenetic alterations have been reported to play critical roles in the development of colorectal cancer (CRC). However, the biological function of the lysine-specific histone demethylase 1B (LSD2/KDM1B) in CRC is not well understood. Therefore, we investigated the characteristics of LSD2 in CRC. We observed significant upregulation of LSD2 in CRC tissue compared to that in normal colorectal tissue. LSD2 promotes CRC cell proliferation and inhibits cell apoptosis through cell cycle regulation, promoting CRC progression both in vitro and in vivo. We found that LSD2 performs these functions by inhibiting the *p53-p21-Rb* pathway. Finally, we found that LSD2 directly binds to p53 and represses p53 expression via H3K4me2 demethylation at the *p53* promoter. Our results revealed that LSD2 acts as an oncogene by binding and inhibiting p53 activity in CRC. Thus, LSD2 may be a new molecular target for CRC treatment.

## INTRODUCTION

Colorectal cancer (CRC) is one of the most common malignant tumors, ranking fourth and second in morbidity and mortality, respectively, among all tumors [1]. An in-depth understanding of the pathogenesis of CRC is important for early diagnosis and treatment. Cell proliferation is a pivotal characteristic of colon cancer [2]. Uncontrolled regulation of the cell cycle is an important cause of cell proliferation [3]. Therefore, one promising strategy for CRC treatment is the identification of molecular targets to regulate the cell cycle [4, 5]. Aberrant epigenetic silencing of tumor suppressor genes or activation of oncogenes are common in cancer [6]. Common epigenetic modifications include DNA methylation and histone covalent modification. Each modification has a context-dependent association with transcriptional activation or repression. For example, histone H3 Lys4 (H3K4) methylation is associated with transcriptional activation, whereas H3K9 methylation is associated with transcriptional repression [7]. Histone methyltransferase (HMTS) can catalyze histone methylation, while methylation can be removed by the catalytic activity of demethylases. The common demethylases include histone demethylase containing the jumonji C (JmjC) domain [8] and FAD-dependent lysine-specific demethylase (LSDs), including LSD1 and LSD2 [9–11]. LSD2 plays important roles in gene silencing, transcription factor activity regulation, and cell cycle regulation in the occurrence and development of many types of tumors [10, 12–14]. Moreover, LSD2 overexpression predicts aggressive tumor biology and poor prognosis [15]. However, while high LSD1 expression has been found in CRC tissues [16], which promotes the proliferation and colony formation of CRC cells [17], the role of LSD2 in CRC biology remains largely unknown.

The results of this study showed that LSD2 is upregulated in CRC tissues. Furthermore, LSD2 overexpression promoted CRC cell proliferation and inhibited cell apoptosis, while LSD2 knockdown dramatically inhibited the cell cycle by causing G1/S arrest and repressed CRC proliferation by regulating the *p53-p21-Rb* pathway both in vitro and vivo. More importantly, LSD2 may transcriptionally repress p53 expression via H3K4me2 demethylation at the *p53* promoter region. These results indicate the important role of LSD2 in CRC progression.

## RESULTS

### LSD2 expression levels in human CRC tissues

**Figure 1:**
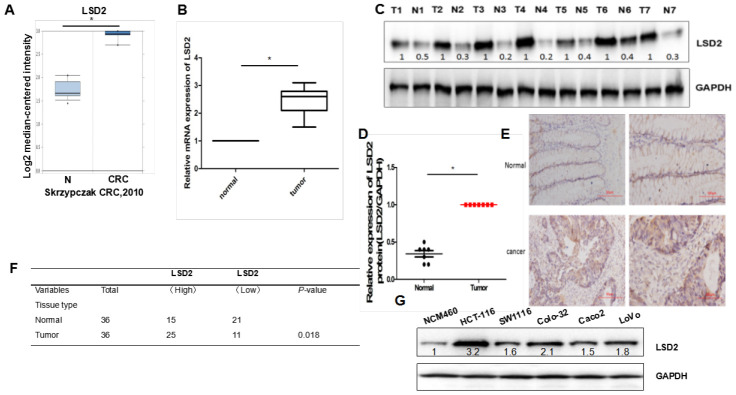
***LSD2* expression levels in human colorectal cancer (CRC) tissue and cells.** (**A**) The ONCOMINE database was used to examine mRNA expression of *LSD2* in CRC tissue. (**B**) Real-time Polymerase chain reaction (PCR) to evaluate mRNA expression of *LSD2* in clinical CRC specimens relative to normal tissues (n=36). Data were expressed as means ± SEM. (**C**, **D**) Western Blot evaluation of LSD2 protein expression in CRC and normal tissue. (**E**) LSD2 protein expression in normal and CRC tissue was analysed by IHC. (**F**) Pearson’s χ2 tests to assess the association between colon tissue type and LSD2 expression by IHC(n=36). (**G**) Western blotting to assess LSD2 expression in CRC cell lines.

We first analyzed LSD2 expression levels in CRC using the Oncomine database. As shown in Figure 1A, compared to that in non-cancerous tissues, LSD2 expression was upregulated in CRC tissues. We further verified LSD2 expression in clinical CRC specimens and analyzed its statistical difference. Consistent with the Oncomine results, LSD2 mRNA was also upregulated compared to that in non-cancerous tissues (Figure 1B). We then used western blotting to measure LSD2 protein expression in CRC. We showed that LSD2 protein was preferentially overexpressed in CRC compared to that in normal tissues (Figure 1C, 1D). Next, immunohistochemistry (IHC) was used to detect the expression of LSD2, which was consistent with previous observations that LSD2 is preferentially overexpressed in CRC tissue (Figure 1E, 1F). We compared LSD2 expression levels between colon cancer cell lines (LoVo, HCT-116, COLO-320, SW1116, and Caco2) and colon cell line NCM460 by western blotting. Results showed that LSD2 expression in CRC cell lines was higher than that in normal colon cell lines (Figure 1G). These results suggest the role of LSD2 in CRC progression.

### Manipulation of LSD2 expression in CRC cells

**Figure 2:**
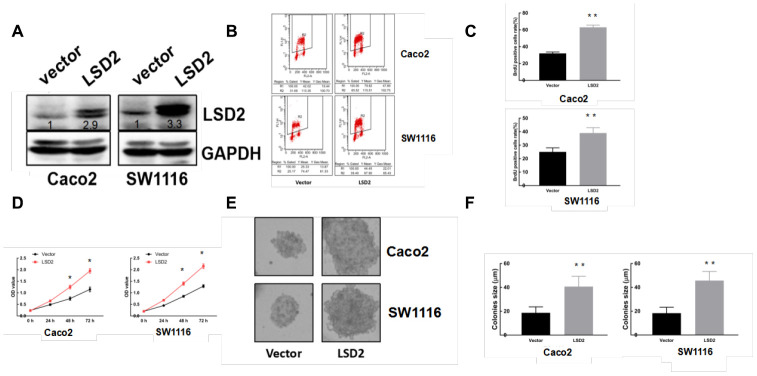
**LSD2 over-expression promotes CRC cell proliferation.** (**A**) Western blot detection of LSD2 and GAPDH (loading control) protein expression in Caco2 and SW1116 cells with control or LSD2. (**B**, **C**) BrdU/PI assay to detect and quantify DNA synthesis in Caco2 and SW1116 cells with control or LSD2. **p < 0.01. (**D**) Cell Counting Kit-8 (CCK8) assay to measure the viability of Caco2 and SW1116 cells with control or LSD2. (**E**, **F**) Colony formation assays and quantification in approximately 500 Caco2 and SW1116 cells with control or LSD2. Data were expressed as means ± SEM, **p < 0.01.

**Figure 3:**
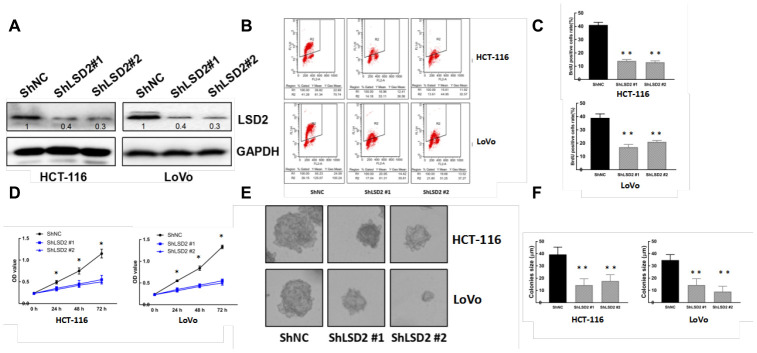
**LSD2 down-regulation inhibits CRC cell proliferation.** (**A**) Western blot showing protein expression of LSD2 and GAPDH (loading control) in HCT-116 and LoVo cells with control or sh-*LSD2*. (**B**, **C**) BrdU/PI assay to detect and quantification of DNA synthesis in HCT-116 and LoVo cells with control or sh-LSD2. **p < 0.01. (**D**) Cell Counting Kit-8 (CCK8) assay to detect the viability of HCT-116 and LoVo cells with control or sh-LSD2. (**E**, **F**) Colony formation assays and quantification in approximately 500 HCT-116 and LoVo cells with control or sh-LSD2. Data were expressed as means ± SEM, **p < 0.01.

We constructed pCDF1-*LSD2* and pCDF1-vector plasmids and established stable LSD2 cell lines Caco2 and SW1116, which were confirmed by western blot (Figure 2A). We also performed lentivirus-mediated knockdown of LSD2 in LoVo and HCT-116 cells using two independent shRNAs to silence, which was also confirmed by western blotting (Figure 3A). We used these cell lines to explore the function of LSD2 in CRC.

### LSD2 promotes CRC cell proliferation both in vitro and in vivo

Given the abnormal LSD2 expression in CRC, we further evaluated the role of LSD2 in CRC proliferation using Cell Counting Kit-8 (CCK8) assays. The results showed that ectopically upregulated LSD2 promoted Caco2 and SW1116 cell growth (Figure 2D), whereas LSD2 knockdown decreased proliferation of HCT-116 and LoVo cells compared to that in the respective controls (Figure 3D). Additionally, BrdU/PI assays showed that, compared to vector cells, LSD2 upregulation increased DNA synthesis in Caco2 and SW1116 cells (Figure 2B, 2C), whereas LSD2 depletion reduced the number of HCT-116 and LoVo cells that incorporated BrdU (Figure 3B, 3C). These results indicated that LSD2 increased DNA synthesis in CRC cell lines. Soft agar colony formation assays showed that LSD2 upregulation increased the colony size and number of HCT-116 and LoVo cells (Figure 2E, 2F), while LSD2 silencing markedly decreased both in HCT-116 and LoVo cells (Figure 3E, 3F). These results suggest that LSD2 upregulation promotes CRC cell proliferation.

**Figure 4:**
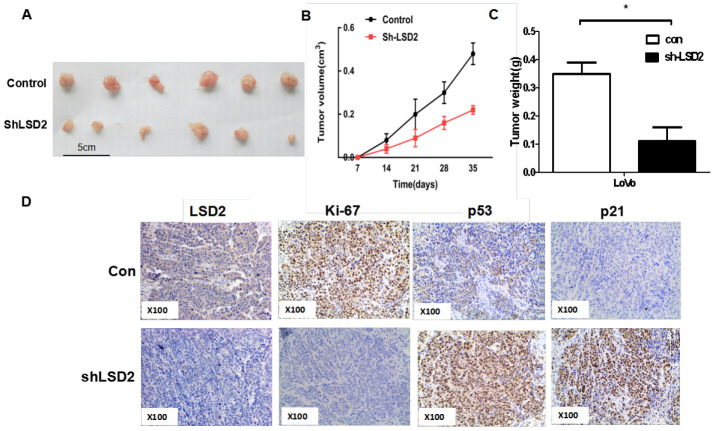
**LSD2 promoted CRC cells proliferation in vivo.** (**A**) Effects of LSD2 down-expression on tumor growth in a xenograft mouse model. Empty vector or sh-LSD2 was transfected into LoVo cells, which were injected in the nude mice (n = 6), and the tumors were obtained at day 35 and was determined by measuring their tumor volume (**B**) and tumor weight (**C**). Data were expressed as means ± SEM. (**D**) The tumor sections were under IHC staining using antibodies against LSD2, p53, p21,ki-67, *P < 0.05.

To further investigate the tumorigenesis function of LSD2 in vivo, a xenograft model of human CRC cells in nude mice was adopted. Sh-LSD2 and vector control LoVo cells were injected subcutaneously into each flank of nude mice. Tumor formation was observed and tumor weight and volume were measured. The sh-LSD2 group exhibited generally smaller tumors (Figure 4A) and displayed less volume (Figure 4B) and weight (Figure 4C) compared to the vector group. This difference was further confirmed following examination of the xenograft by IHC: the tumors developed from sh-LSD2 cells displayed lower Ki-67 staining than the control group (Figure 4D). Taken together, these results indicate that LSD2 plays a vital role in the tumorigenicity and tumor growth of CRC.

### LSD2 reduced apoptosis and induced cell cycle progression to promote CRC cell proliferation

**Figure 5:**
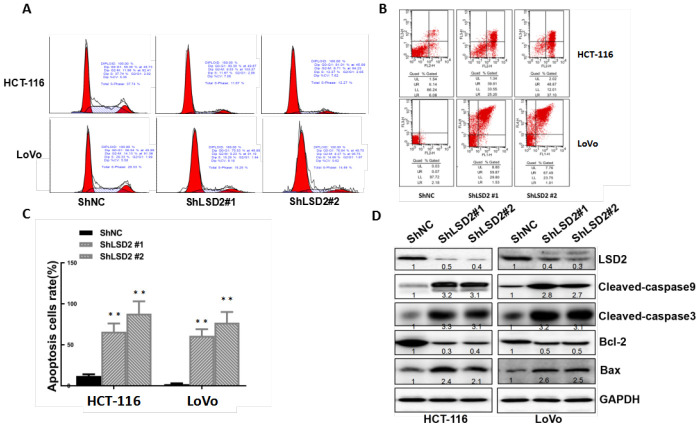
**LSD2 promotes proliferation by inducing G1-S arrest and reducing apoptosis in colorectal cancer (CRC) cells.** (**A**) HCT-116 and LoVo cells stably expressing vector or sh-LSD2#1 or sh- LSD2#2. The percentage of cells in G0/G1, S. or G2/M phases was tested using a subG1 assay and flow cytometry. (**B**) Apoptotic rates of HCT-116 and LoVo cells stably expressing vector or sh-LSD2#1 or sh-LSD2#2 measured by flow cytometry in annexin V/PI assays. LR, early apoptotic cells; UR, terminal apoptotic cells. Values represent the means ± SD. (**C**) Quantitative analysis of DNA apoptosis. Data were expressed as means ± SEM. **p < 0.01. (**D**) Bcl-2, BAX, CL-caspase 3, and CL-caspase 9 protein expression measured by Western blot with GAPDH protein as the loading control.

Cell cycle dysregulation is one of the most important reasons for cell proliferation in cancer. Flow cytometry was used to explore whether LSD2 promoted CRC proliferation by regulating cell cycle progression. We observed a dramatic increase in G1/S-phase cell cycle arrest in HCT-116 and LoVo cells with sh-LSD2. We also observed a reduced population of S-phase cells (Figure 5A). Annexin V/PI assays to test apoptosis rates showed higher percentages of early and late apoptotic cells in HCT-116 and LoVo cells with sh-LSD2 compared to those in the control cells (Figure 5B, 5C). Moreover, western blot assays showed decreased levels of the apoptosis inhibitor Bcl-2 and significantly increased levels of CL-caspase 3/CL-caspase 9 and the apoptosis sensor BAX in HCT-116 and LoVo cells with sh-LSD2 (Figure 5D). These results indicate that LSD2 regulates the cell cycle in CRC.

### LSD2 epigenetically silenced *p53* transcription through LSD2-mediated H3K4me2 demethylation

**Figure 6:**
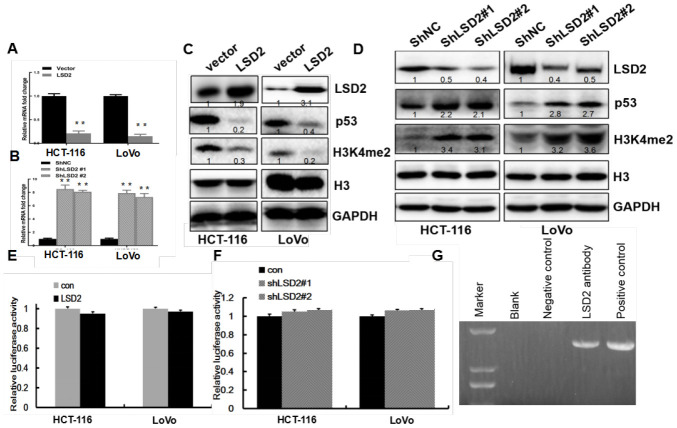
**LSD2 inhibits p53 expression via demethylation of H3K4me2 through binding to the *p53* promoter.** (**A**, **B**) Real-time PCR assays to detect *p53* mRNA expression for vector, LSD2, ShNC, and Sh-LSD2 in HCT-116 and LoVo cells. (**C**, **D**) p53 and H3K4me2 protein expression measured by Western blot for vector, LSD2, and ShNC in HCT-116 and LoVo cells. (**E**, **F**) *P53* promoter activity in vector, LSD2, ShNC and Sh-LSD2 in HCT-116 and LoVo cells measured by luciferase assay. Data were expressed as means ± SEM. (**G**) ChIP assay in LoVo cells showing the interaction between LSD2 and H3K4me2 at the *p53* promoter. **p < 0.01.

The p53 pathway is an important cell cycle regulator [18]. To explore the effect of *LSD2* on p53 expression in CRC cells, real-time PCR was performed to detect changes in *p53* mRNA expression in HCT-116 and LoVo cells with sh-LSD2. The results revealed increased *p53* expression in sh-LSD2 HCT-116 and LoVo cells compared to that in the control cells (*p* < 0.01, Figure 6B). Moreover, ectopic expression of *LSD2* downregulated *p53* expression (Figure 6A). Western blots revealed increased p53 protein levels in HCT-116 and LoVo cells with sh-LSD2 cells (Figure 6D), while LSD2 overexpression decreased p53 protein levels compared to those in control cells (Figure 6C).

LSD2 affects target genes by regulating H3K4me2 methylation levels [7]. To determine how LSD2 regulates p53 expression, we first measured histone 3 protein methylation levels in HCT-116 and LoVo cells. Compared to the control group, LSD2 upregulation inhibited H3K4me2 methylation, while LSD2 knockdown increased H3K4me2 methylation (Figure 6C, 6D).

This evidence suggested that LSD2 could regulate p53 expression by directly binding to *p53*. To verify this hypothesis, we performed CHIP assays in LoVo cells, finding that anti-LSD2 antibodies directly immunoprecipitated the *p53* promoter (Figure 6G). We performed luciferase assays to determine whether LSD2 could activate the *p53* promoter. The results showed that *p53* promoter activity did not change significantly regardless of LSD2 overexpression (Figure 6E) or in sh-LSD2 CRC cells (Figure 6F).

In summary, our findings indicate that LSD2 represses p53 expression through demethylation of H3K4me2 at the *p53* promoter.

### LSD2 downregulated p53 expression driving the cell cycle through the *p53-p21-Rb* pathway

**Figure 7:**
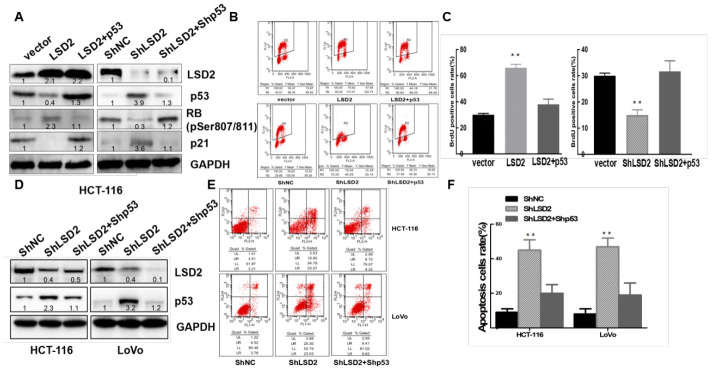
**LSD2 regulates colorectal cancer (CRC) cell proliferation and apoptosis through p53. ShRNA mediates p53 knockdown in HCT-116 LSD2-downregulated cell lines, while p53 over-expression in HCT-116 LSD2-upregulated cell lines was observed.** (**A**) Protein expression of p53, p21, pRb and LSD2 measured by Western blot among the CRC cell lines mentioned above. (**B**) BrdU/PI assays were performed to measure DNA synthesis in the indicated CRC cell lines. (**C**) Quantitative analysis of DNA synthesis. (**D**) Western blot showing p53 and LSD2 protein expression in the p53 LSD2 double-downregulated HCT-116 and LoVo cell lines. (**E**) Flow cytometry detection of the apoptotic rates of the indicated CRC cell lines. (**F**) Quantitative analysis of DNA apoptosis. Data were expressed as means ± SEM. **p < 0.01.

p53 plays a central role in the induction of G1/S phase arrest by inducing p21, an inhibitor of cyclin-dependent kinases (CDKs), leading to dephosphorylation and activation of cell cycle repressor retinoblastoma (Rb) [19]. To confirm whether LSD2 promoted CRC proliferation through the *p53-p21-Rb* pathway, we overexpressed p53 in HCT-116 cells stably expressing LSD2 (Figure 7A). Western blotting revealed decreased levels of p53 and p21 and increased levels of RB(pSer807/811) in HCT-116 cells with LSD2. The upregulation of p53 can reverse the decrease in p21 and the increase in RB(pSer807/811). We then knocked down both LSD2 and p53 in HCT-116 cells. The expression of p53 and p21 increased, while the expression of RB decreased, which was caused by the knockdown of LSD2, and the effect could be reversed by downregulation of p53 (Figure 7A). BrdU/PI assays revealed that p53 upregulation counterbalanced the promotion of proliferation mediated by LSD2 (*p* < 0.01) (Figure 7B, 7C).

We then knocked down p53 in stable HCT-116 and LoVo cells expressing sh-LSD2 (Figure 7D). Annexin V/PI assays showed that knockdown of both LSD2 and p53 partially decreased CRC apoptosis compared to that of LSD2 downregulation alone (Figure 7E, 7F).

We also detected the expression of p53 and p21 by IHC in xenograft animal studies and found that the expression of p53 and p21 elevated in tumors developed from sh-LSD2 cells (Figure 4D), suggesting that LSD2 affects the cell cycle through the p53-p21-Rb pathway in vivo.

These results indicated that LSD2 regulated the CRC cell cycle partly through the p53-p21-Rb pathway.

## DISCUSSION

From a genomic standpoint, CRC is not a single disease but a heterogeneous group of malignancies arising within the colon. Genomic analysis of CRC provides important prognostic and predictive information for clinicians [1]. For example, CRC patients with KRAS, NRAS, or BRAF mutations do not benefit from anti-epidermal growth factor receptor (EGFR) therapies such as cetuximab or panitumumab [20–23]. Thus, understanding the molecular events involved in the development of CRC is important for the accurate treatment of CRC.

Previous studies have suggested the role of epigenetic post-translational modifications in CRC progression [24–26]. Histone methylation is one of the most important epigenetic regulations, representing a fundamental epigenetic mechanism underlying eukaryotic gene embryotic development, cellular proliferation. DNA proofreading and repair, chromatin remodeling, transcription regulation, etc. [27, 28]. Two families of histone demethylases (KDMs) have been discovered: flavin-dependent lysine-specific demethylases and JmjC domain-containing KDMs. The flavin-dependent KDM family includes LSD1 (KDM1A/AOF2) and LSD2 (KDM1B/AOF1) [12]. LSD1 has been shown to be overexpressed in colorectal cancer and to promote the growth and invasion of colorectal cancer [16, 17]. Although the function of LSD2 in the biology of other cancers such as breast cancer has been described in detail [15], the expression and function of LSD2 in CRC has not been reported until now.

We observed LSD2 upregulation in CRC tissue and further evaluated the effect of LSD2 in CRC cell apoptosis and proliferation. We found that LSD2 upregulation strongly inhibited CRC cell apoptosis and enhanced cell proliferation, while LSD2 depletion significantly promoted CRC cell apoptosis and attenuated cell proliferation, indicating the involvement of LSD2 in the regulation of CRC progression. Targeting *LSD2* may provide new ideas and effective treatments for CRC.

We further explored the molecular mechanisms by which LSD2 regulates CRC proliferation and apoptosis. The tumor suppressor activity of p53 lies in its ability to induce cell cycle arrest and apoptosis in oncogenic cells [29]. Although the effect of p53 on the role of the LSD family in colorectal cancer has been studied before, it remains controversial. Although it has been reported that LSD1 can regulate p53 by removing both monomethoxylation (k370me1) and dimerization (k370me2) at k370 [30], it has been reported that the expression of p53 is not related to LSD1 in CRC tissue [17].In our study, p53 was correlated with LSD2 function in CRC. We showed that LSD2 overexpression downregulated the protein levels of p53, while LSD2 knockdown upregulated p53. Moreover, double knockdown of LSD2 and p53 reversed the effect of LSD2 on proliferation and apoptosis in CRC. These findings indicate that LSD2 induces tumor proliferation and inhibits apoptosis via p53 expression. We found that the knockdown of LSD2 caused G1/S arrest in CRC cell lines, and the effect of p53 on cell cycle arrest is mainly dependent on the p53-p21-Rb pathway [31]. Therefore, we detected the expression of p21 and RB in this pathway, and found that LSD2 regulates the cell cycle distribution of CRC through the *p53-p21-Rb* pathway in vivo and in vitro, which promotes the proliferation and growth of CRC.

We further showed that LSD2 is an H3K4me2 demethylase that specifically regulates histone H3K4 methylation at the *p53* promoter. While genome-wide mapping indicates that LSD2 associates predominantly with gene bodies [10], our results showed that LSD2 binds to the promoter region of *p53* in CRC. A specific function of LSD2 is to maintain low levels of H3K4 methylation [10].

Previous studies showed that LSD2 alone had significant H3K4 demethylase activity on nucleosomal substrates, a clear distinction from that of LSD1 [10]. Our results showed that the depletion of endogenous LSD2 resulted in increased H3K4me2. However, rather than functioning as a transcription activator, LSD2 is instead a repressor of p53. The LSD2 repression of p53 in CRC is likely dependent on its H3K4 demethylation activity.

Our results demonstrated the important role of LSD2 in CRC progression. First, we observed LSD2 overexpression in CRC samples. Second, LSD2 promoted CRC proliferation and reduced apoptosis both in vitro and in vivo. Third, *LSD2* regulates the cell cycle distribution of CRC through the *p53-p21-Rb* pathway in vivo and in vitro. Finally, LSD2 directly binds to the *p53* promoter to downregulate p53 through H3K4me2 demethylation. *LSD2* may be a new molecular target for CRC treatment.

## MATERIALS AND METHODS

### Cell culture and reagents

LoVo, HCT-116, COLO-320, SW1116, and Caco2 cells were obtained from the American Type Culture Collection (ATCC) and cultured in Dulbecco’s modified Eagle’s medium (DMEM) or RPMI-1640 (COLO-320) supplemented with 10% fetal bovine serum at 37°C. Normal colonic epithelial cells (NCM460) were cultured in F12 supplemented with 20% fetal bovine.

Antibodies against LSD2 (ab193080 for CHIP and WB, ab234863 for IHC), p53 (ab32389), H3K4me2 (ab7766), p21 (ab227443) and Ki-67(ab15580) were purchased from Abcam (Cambridge, MA, USA), while CL-caspase 9, CL-caspase 3, BAX, Bcl-2, Phospho-Rb (Ser807/811)(9308) and GAPDH antibodies were purchased from Cell Signaling (Danvers, MA, USA).

### Stable cell lines

Stable cell lines overexpressing LSD2 were established using the pCDF1 lentivirus system (System Biosciences, SBI) as previously described [32].

Two different *LSD2* shRNAs and controls were obtained from SABiosciences (Germantown, MD, USA) and reverse transfected into HCT-116 and LoVo cells. The cells were then sorted by flow cytometry after selection in 800 μg/mL G418 for 4 weeks and confirmed by western blotting.

### Clinical samples and analysis of the cDNA microarray database

The expression of *LSD2* mRNA in CRC was first examined using the Oncomine database (https://www.oncomine.org/) [33], which compares the expression of genes in different tissues based on previous research results [34].

Human samples were obtained from colorectal carcinoma patients without chemotherapy or radiotherapy after receiving approval from the Ethical Committee of the Medical Faculty of Fujian Medical College (Fuzhou, PR China) [32].

IHC was performed as previously described [35] using antibodies against LSD2.

### Western blot analysis

Proteins were extracted from cells and tissues and measured by western blotting using the antibodies mentioned above, as previously described [36].

### Cell proliferation assay

BrdU/PI assays were performed as previously described [36]. We also performed cell counting Kit-8 (CCK8) cell proliferation assays. Briefly, 2,000 cells were seeded in 96-well plates for 1 week before 10 μL CCK8 solution was added to each plate and incubated at 37°C for 2 hours. The absorbance at 450 nm of each plate was measured and compared.

### Colony formation assay

A total of 500 cells were seeded into 6-well plates containing 1% noble agar. The medium was replaced every 3 days for 2 weeks before colony formation was observed under a microscope.

### Cell cycle analysis and annexin V/propidium iodide detection

PI staining and annexin V/PI staining were performed to assess changes in phase distribution based on DNA content as well as apoptosis rates after manipulating LSD2 expression levels by flow cytometry (FACS Vantage; BD Biosciences), as described previously [36].

### Real-time polymerase chain reaction (PCR) assays for *LSD2* and *p53*

Total RNA extraction, complementary DNA (cDNA) synthesis, and real-time PCR assays were performed as described previously [19] using the following primer sequences:*LSD2*-F (5′-AACCGAACCTAGTCCCAAAG-3′) and *LSD2*-R (5′-GGCTATCTGTGGAGTAAGCT-3), *p53*-F (5′-ACCTATGGAAACTACTTCCTGAAA-3′) and *p53*-R (5′-CTGGCATTCTGGGAGCTTCA-3′), GAPDH-F (5′-GGAGCGAGATCCCTCCAAAAT-3′), and GAPDH-R (5′-GGCTGTTGTCATACTTCTCATGG-3′).

### Luciferase assay

The transcriptional activity of the *p53* promoter was measured using this assay, as previously described [35]. The *p53* promoter sequence was cloned into a pGL3-basic luciferase reporter vector and co-transfected with or without *LSD2* (sh-*LSD2*).

### Chromatin immunoprecipitation

An EZChIP™ ChIP Kit (Millipore, Bedford, MA, USA) was used to perform this assay. We first sonicated chromatin DNA into fragments ranging in size from 200 to 500 bp, using RNA-polymerase II (binding to GAPDH) as a positive control and normal mouse immunoglobulin G (IgG) as a negative control. The chromatin was then immunoprecipitated with anti-LSD2 (1:1000). The LSD2 antibodies for the ChIP assays were purchased from Cell Signaling Technology. The GAPDH primers were provided in the kit, and the *p53* primers used were *p53*-CF (5′-ATGTTAGTATCTACGGCACCAG-3′) and *p53*-CR (5′-CAGCCCGAACGCAAAGTG-3′)./

### Animal studies

Female BALB/c NOD mice were bred to 4–5 weeks of age. CRC cells (5×10^6^) in 50 μL DMEM were mixed with 50 μl ice-cold Matrigel and then subcutaneously injected into the nude mice. We measured tumor volumes and dimensions every 7 days until day 35, when the animals were sacrificed. The tumor xenografts were then weighed and bisected, analyzed by IHC for LSD2, p53, p21, and Ki-67. Our study was approved by the Animal Experimentation Committee of the Central Institute for Experimental Animals, Fujian Medical University.

### Statistical analysis

Data are expressed as mean ± standard error of the mean (SEM). Analysis of variance (ANOVA) and Student’s t-tests were used for comparisons between groups. SPSS version 13.0 was used for statistical analysis. *p* < 0.05 was considered statistically significant.
